# Ionizing radiation improves skin bacterial dysbiosis in cutaneous T-cell lymphoma

**DOI:** 10.3389/fimmu.2024.1520214

**Published:** 2024-12-24

**Authors:** Lauren P. Chrisman, Yanzhen Pang, Madeline J. Hooper, Greeshma Rajeev-Kumar, William Q. Nguyen, Stefan J. Green, Patrick C. Seed, Hua Liang, Bharat B. Mittal, Yasmin Hasan, Joan Guitart, Ralph R. Weichselbaum, Michael B. Burns, Xiaolong A. Zhou

**Affiliations:** ^1^ Department of Dermatology, Northwestern University, Feinberg School of Medicine, Chicago, IL, United States; ^2^ Department of Radiation and Cellular Oncology, University of Chicago Medicine, Chicago, IL, United States; ^3^ Genomics and Microbiome Core Facility, Rush University Medical Center, Chicago, IL, United States; ^4^ Stanley Manne Children’s Research Institute, Ann and Robert H. Lurie Children’s Hospital of Chicago, Chicago, IL, United States; ^5^ Department of Pediatrics, Northwestern University, Feinberg School of Medicine, Chicago, IL, United States; ^6^ The Ludwig Center for Metastasis, University of Chicago Medicine, Chicago, IL, United States; ^7^ Department of Radiation Oncology, Northwestern University, Feinberg School of Medicine, Chicago, IL, United States; ^8^ Department of Biology, Loyola University Chicago, Chicago, IL, United States

**Keywords:** cutaneous T-cell lymphoma, microbiome, radiation, radiotherapy, total skin electron beam therapy, skin cancer, lymphoma

## Abstract

**Introduction:**

Cutaneous T-cell lymphoma (CTCL) is closely associated with the host microbiome. While recent evidence suggests that shifts in specific bacterial taxa are associated with response to UV-B, a form of non-ionizing radiation, the impact of ionizing radiation (IR) has not been investigated.

**Methods:**

16S rRNA and *tuf* gene amplicon sequencing were performed on DNA extracted from swabs of lesional/non-lesional skin of 12 CTCL patients before/after TSEBT or local IR and from 25 matched healthy controls (HC). Microbial diversity and taxonomic profiles were analyzed.

**Results:**

Radiation exposure increased CTCL skin α-diversity to levels approximating HC. TSEBT appeared to carry the greatest effect compared to local IR. Both α and β-diversity differed significantly post versus pre-IR for TSEBT, but not for local IR. IR was associated with decreases in known pathogenic bacteria such as *Streptococcus* and *S. aureus* and increases in healthy commensal bacteria such as *Anaerococcus, Bifidobacterium* and commensal staphylococci including *S. pettenkoferi.* Substantially more taxa shifts were seen with TSEBT versus local IR.

**Discussion:**

IR not only eliminates CTCL lesions via induction of apoptosis, but also facilitates skin barrier restoration and recolonization of bacterial taxa associated with a healthy skin microbiome. Local IR does not have as strong an effect on the skin microbiome as TSEBT. As skin microbiota act as immunomodulators with local and potentially systemic influence, TSEBT may also improve CTCL lesions via global effects on the skin microbiome. Future larger-scale studies are required to fully elucidate the relationship between cutaneous microbes and IR treatment in CTCL.

## Introduction

Cutaneous T-cell lymphoma (CTCL) encompasses a heterogenous group of non-Hodgkin T-cell lymphomas characterized by malignant T-cells in the skin (1). Evidence increasingly suggests an intimate connection between the host microbiome and CTCL disease pathogenesis. Although the etiology of CTCL remains unknown, skin bacteria can fuel disease progression (2–4). Skin, nasal and gut microbiome changes correlate with CTCL disease progression and severity (5–7). Additionally, broad-spectrum antibiotics may reduce tumor burden in some patients (8).

Ionizing radiation (IR) is one of the most effective treatments for CTCL (9). Local IR is often used for solitary lesions, whereas total skin electron beam therapy (TSEBT), a procedure which delivers IR to the entire skin surface, is typically utilized in patients with more diffuse disease (9). We previously demonstrated that narrowband ultraviolet B (nbUVB), a form of non-ionizing radiation utilized for treatment of CTCL, alters the skin and gut microbiomes of CTCL patients, and others have shown that the skin microbiome modulates the effect of UV on inflammation (10–13). Moreover, the skin microbiome of nbUVB responders, but not non-responders, has increased microbial diversity and shifts in the relative abundance of certain bacterial taxa, including increased pre-treatment abundances of *S. capitis* and *S. warneri*, and decreased post-treatment *S. aureus* and *S. lugdunensis*, that may be predictive of response to nbUVB treatment (10). Given these findings, we hypothesize that similar shifts may be identified with IR, which remains unexplored.

Prior studies have cross-sectionally examined skin microbial changes with respect to radiation injury, but none to our knowledge have addressed the longitudinal changes associated with palliative IR (14). For example, cancer patients experiencing IR-induced dermatitis have significantly reduced bacterial diversity and overrepresentation of *Staphylococcus*, *Pseudomonas*, and *Stenotrophomonas* when compared to healthy controls (15). Furthermore, patients experiencing radiation-induced skin injury have significantly different relative abundances of bacterial taxa when compared to healthy controls (16).

Herein, we performed a longitudinal study of the skin bacterial microbiome of CTCL patients receiving IR and compared them with matched healthy controls. This knowledge may improve our understanding of the skin microbiome in CTCL and the effects of IR on the skin microbiome.

## Materials and methods

### Participants

Ethical approval was obtained from the Northwestern University Institutional Review Board (IRB) (STU00209226) & University of Chicago IRB (IRB22-0595). Patients were consented and enrolled at the Northwestern University Cutaneous Lymphoma clinic and University of Chicago Radiation Oncology clinic between 2019 and 2023 in compliance with the Declaration of Helsinki. Demographic/clinical data and lesional and contralateral non-lesional skin samples were collected from 12 patients with biopsy-confirmed CTCL (**Supplementary Table S1**). Six participants received local IR, and 6 patients received TSEBT (**Supplementary Table S2**). Eleven patients were concurrently on topical steroids and 8 on systemic therapy (i.e., acitretin and interferon) but no new treatments were introduced within 6 months prior to or during the study interval and any concurrent therapies were present for at least 6 months prior to IR. Additionally, there were no changes in diet, bathing habits, or non-CTCL medications based on chart review and survey data collected during the study interval. Patients who utilized antibiotics within the 4 weeks prior to collections were excluded. Healthy controls (HC) were comprised of 25 volunteers without CTCL or other active skin disease (**Supplementary Table S3**).

### Sample collection and DNA extraction

Skin samples from 12 CTCL patients and 25 HC were obtained through sterile swabs. All specimens were placed immediately in sterile cryovials and stored at -80°C until DNA extraction. Genomic DNA was extracted using a Maxwell 16 LEV Blood DNA Kit (Promega, Madison, WI) implemented on a Maxwell 16 Instrument, following the manufacturer’s instructions with minor modifications: a lysozyme incubation (10 ng/μl lysozyme; Thermo Fisher Scientific, Waltham, MA) for 30 minutes at 37°C and bead beating (40 seconds at 6 min/sec) using a FastPrep-24 System (MP Biomedicals, Irvine, CA). Homogenized samples were transferred to the Maxwell cartridges for final DNA purification.

### 16S rRNA amplification and sequencing

Genomic DNA was prepared for sequencing using a two-stage amplicon workflow and targeting the V4 variable region of microbial 16S rRNA genes as described previously (10, 17, 18).

### Basic processing

Sequencing resulted in a total of 9,257,316 reads with an average of 31,275 reads per sample. Forward (F) and reverse (R) reads were trimmed using cutadapt v3.5 to remove primer sequences (19). Processing, decontamination, and filtering were performed in a matter identical to our previous work examining nbUVB and the CTCL skin microbiome (10, 20–23). Following QC processing, decontamination, and filtering, there were a total of 5,125,206 merged read pairs with an average of 18,982 per sample. To maximize data retention, while removing uninformative patient samples, only samples with minimum 1000 reads following processing were retained. Patient samples were paired across disease status (lesional, non-lesional) and time (pre-IR, post-IR).

### Tuf2 amplicon next generation sequencing

Genomic DNA was PCR amplified with primers CS1_tuf2-F (ACACTGACGACATGGTTCTACAACAGGCCGTGTTGA ACGTG) and CS2_tuf2-R (TACGGTAGCAGAGACTTGG TCTACAGTACGTCCACCTTCACG) targeting the *Staphylococcus tuf* gene (24, 25). Amplicons were generated using a two-stage PCR amplification protocol, as previously described and utilized in our previous work (10, 17).

### Tuf2 amplicon processing

Tuf2 sequencing generated a total of 10,289,743 reads with an average of 34,880 raw reads per sample. Primer trimming and denoising were accomplished using the same procedure as above for the 16S amplicon data with the following modifications: during filtering and trimming, the maxEE for all reads was set to 3,3 due to their increased length, read merging used default parameters, and the read length cutoff window range was 460-470 nucleotides for merged read pairs. Following processing there were a total of 3,914,382 reads retained for an average sample read count of 13,269. ASVs were taxonomically annotated using BLAST alignments against NCBI prokaryotic (nr) refseq database (online access 10 April 2023). Only sequences that were annotated as *Staphylococcus* were retained.

### Statistical analysis

The samples in the cleaned ASV table were visually evaluated using phyloseq v1.42.0 (26). α-diversity metrics were generated using the ASV table rarefied to 1000 sequences. Differences in α-diversity between patient sets were calculated using Wilcoxon rank-sum non-paired tests from the stats R package while differences within patient-matched samples were calculated using Pairwise Wilcoxon rank-sum tests. β-diversity metrics were generated using the rarefied ASV table (27). Principal coordinate analysis (PCoA) with Bray-Curtis dissimilarity was performed to identify β-diversity using an ADONIS2 method with default parameters (28).

Differential abundance analysis was conducted by DESeq2 v1.44.0 using the non-rarefied, CLR-transformed ASV table, with ASVs removed if they had less than 4 counts or a prevalence below 10% across the sample set (29). A linear model was implemented within the approach to compare abundance of taxa among different groups. Significant ASVs were only considered if they achieved a false discovery rate (BH-FDR)-adjusted p-value of <0.05 (q-value).

The post-process tuf2 ASV and taxa table was filtered using phyloseq v1.38 (26). To identify the most abundant *Staphylococcus* species, data were centered log-ratio (CLR) transformed and the final species-level table including only species present at 10% abundance or greater was used for statistical analysis. Differences in abundance between patient groups were calculated using Wilcoxon rank-sum non-paired tests from the stats R package while Pairwise Wilcoxon rank-sum tests were used to calculate differences between patient-matched samples (27). Differential abundance analysis was conducted using the CLR transformed abundance table. A linear model as described above was implemented to compare abundance of the top *Staphylococcus* species among different groups. Significant ASVs were only considered if they achieved an FDR of <0.05.

## Results

### Patient and IR characteristics

The median age of CTCL patients was 56 years (range 21-77) and 9 patients (75%) were male. Ten (83%) patients had mycosis fungoides, and 2 had other CTCL types. Median time between pre-IR sample collection and IR was 20 days; median time between IR and post-IR sample collection was 30 days. Modified Severity Weighted Assessment Tool (mSWAT) decreased by 25.3 points, on average, after IR. Patients receiving local IR received an average of 9.5 Gray over 1-12 fractions; patients receiving TSEBT received an average of 20 Gray over 6-16 fractions. There were no significant differences between age, sex, race, Fitzpatrick skin type (FST), CTCL subtype, stage, or non-IR therapies between patient’s receiving local IR versus TSEBT (p>0.05) (**Supplementary Table S4**). There were no statistically significant differences between HC and patient age, sex, race, or comorbidities (p>0.05) (**Supplementary Table S5**).

### IR is associated with increased microbial diversity and greater microbial shifts in non-lesional skin

**Figure 1:**
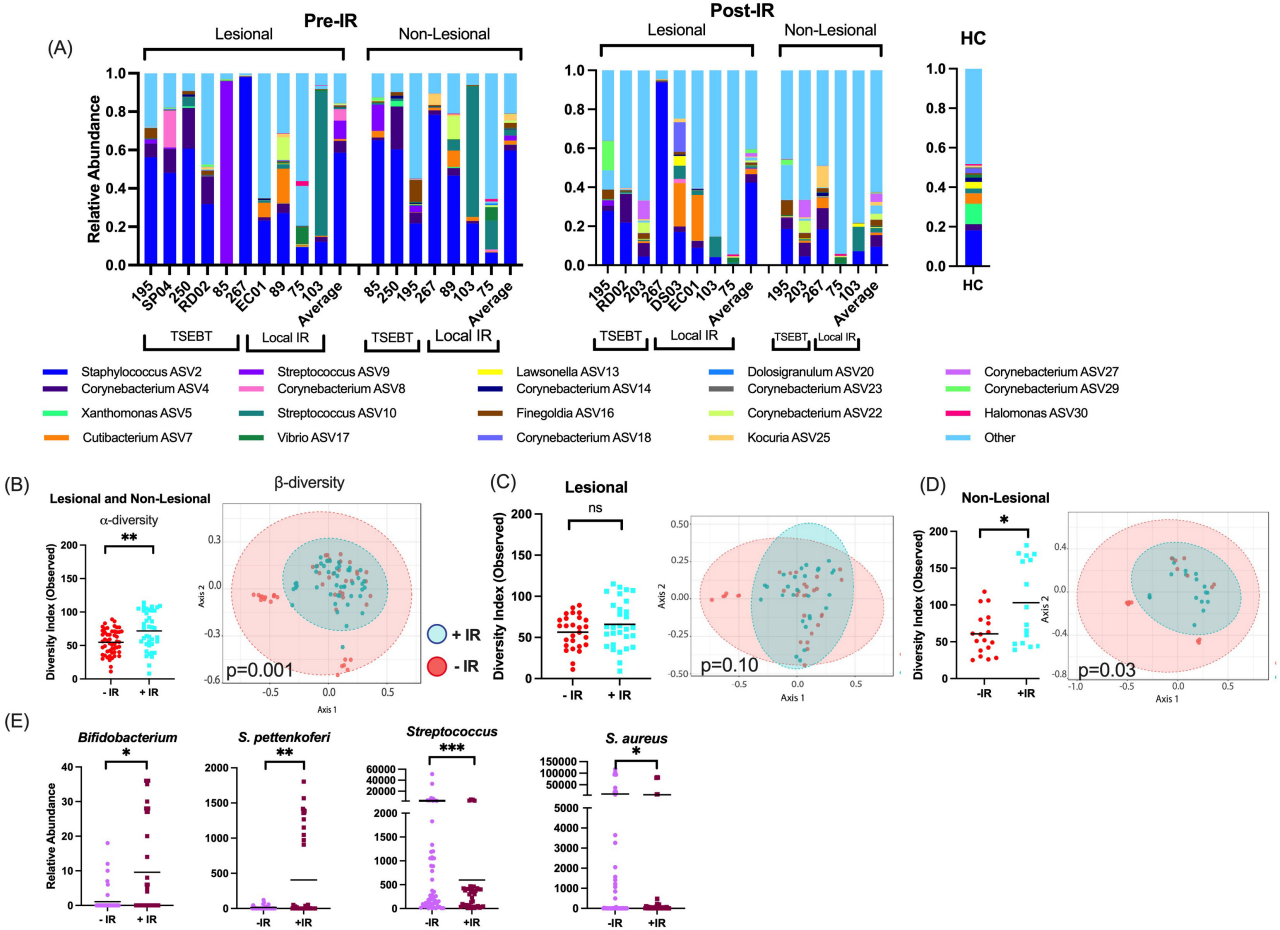
Taxon-by-taxon and α- and β-diversity analyses for skin exposed to any form of ionizing radiation (IR). **(A)** At the taxonomic level of genus, *Staphylococcus, Corynebacterium*, and *Streptococcus* were the most prevalent and abundant genera amongst all samples. Bar charts indicate the relevant abundance of the 19 most abundant genera, remaining genera were grouped as “other.” **(B)** For any skin (both lesional & non-lesional) exposed to any form of IR, α-diversity is higher with IR exposure, and β-diversity is significantly different between the two communities. **(C)** For only lesional skin exposed to IR, α- and β-diversities are not significantly different between skin with and without IR exposure. **(D)** For non-lesional skin exposed to any form of radiotherapy, α-diversity and β-diversity were significantly different between skin with and without IR exposure. **(E)** Taxa-by-taxa analyses with and without IR exposure reveals significantly different relative abundances of specific bacterial taxa. *p <0.05; **p<0.01; ***p<0.001; ns, not significant.

Across all individuals, *Staphylococcus* was the most abundant genus pre- and post-IR, followed by *Corynebacterium* and *Streptococcus* (**Figure 1A**).

We first analyzed any skin (both lesional and non-lesional) exposed to any form of IR (both local IR and TSEBT). Phylogenetic diversity (α-diversity) was significantly higher with IR exposure (Observed p=0.0019), and community structure (β-diversity) differed significantly between skin with and without IR exposure (p=0.001) (**Figure 1B**). Next, we analyzed only lesional skin exposed to IR. In this analysis, there were no significant differences in α- or β-diversity between lesions with and without exposure (**Figure 1C**). Finally, we analyzed only non-lesional skin exposed to IR. Phylogenetic diversity was significantly higher with IR exposure (p=0.02), and β-diversity differed significantly between non-lesional skin with and without IR exposure (p=0.03) (**Figure 1D**).

When observing changes to taxa, healthy commensal bacteria such as *Bifidobacterium* and coagulase negative species *S. pettenkoferi* were significantly more abundant in skin with IR exposure, and known pathogenic bacteria such as *Streptococcus* and *S. aureus* were either significantly lower (p<0.01, q<0.05) or trended lower (p<0.05, q>0.05), respectively, in skin with IR exposure. (**Figure 1E**). Comparatively, very few bacteria with pathogenic potential were increased post-IR exposure. These included *Trueperella* and *Porphyromonas* which have only been reported to cause skin infections in rare opportunistic cases (30, 31).

### TSEBT, but not local IR, increases bacterial diversity and modifies bacterial community structure

**Figure 2:**
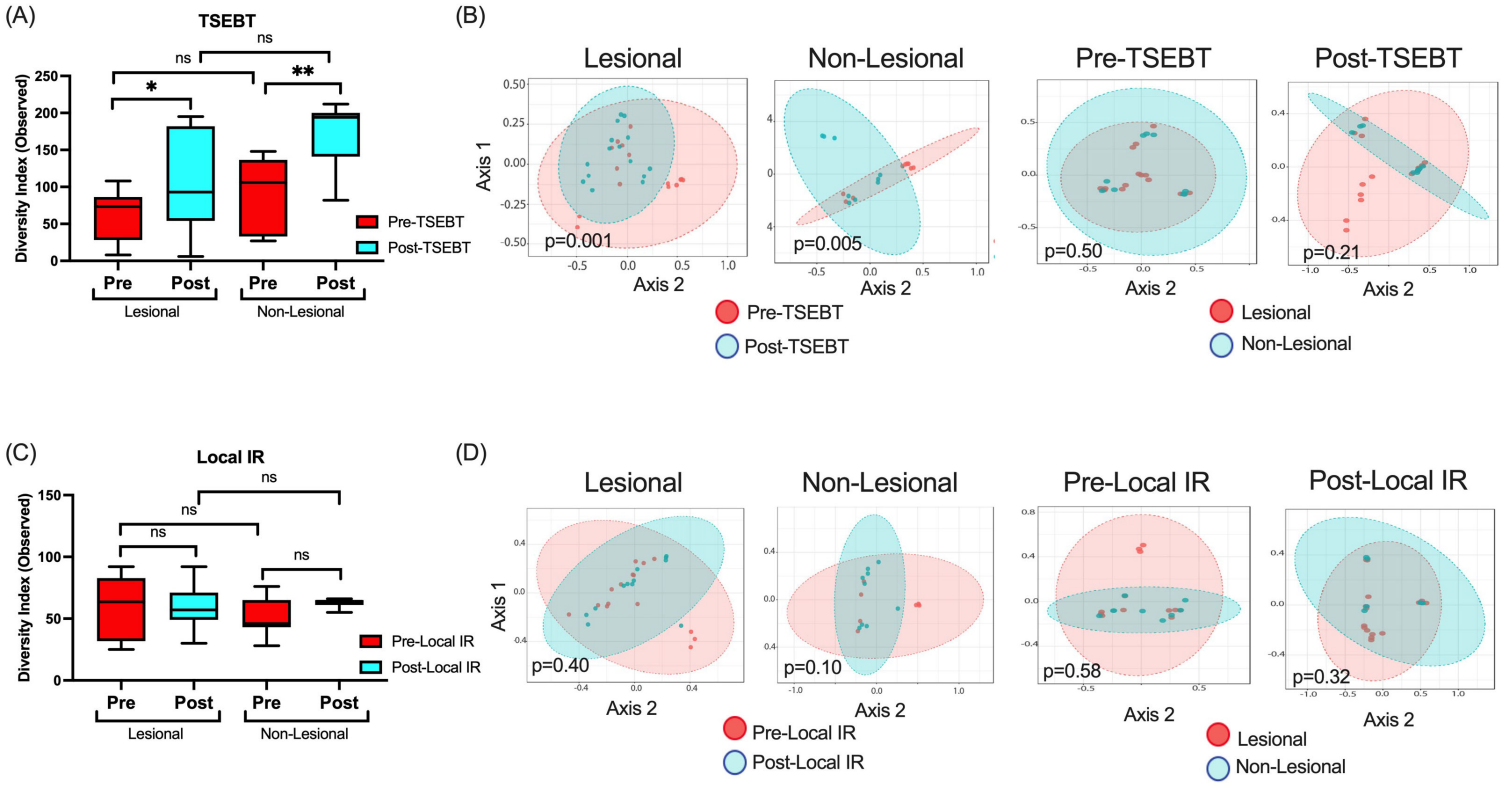
α- and β-diversity analyses for the TSEBT and local-IR study groups analyzed. **(A)** Analyses of both lesional and non-lesional skin before and after TSEBT demonstrates higher α-diversity post-TSEBT for both groups. There was no significant difference in α-diversity between lesional and non-lesional skin at either pre-TSEBT or post-TSEBT time points. **(B)** β-diversity for lesional and non-lesional skin reveals different microbial communities for pre- versus post-TSEBT but not between lesional and non-lesional skin at pre-TSEBT and post-TSEBT timepoints. **(C)** Analyses of α-diversity amongst local IR samples demonstrate no change with treatment or between lesional and non-lesional samples. **(D)** There were no differences in β-diversity between pre and post-local IR for either lesional or non-lesional samples, or between lesional and non-lesional samples at either pre-local IR or post-local IR timepoints. *p <0.05; **p<0.01; ns, not significant.

Next, we compared α- and β-diversity between pre-TSEBT and post-TSEBT and between lesional and non-lesional skin. α-diversity was significantly higher post-TSEBT compared to pre-TSEBT in both lesional (Observed p=0.024) and non-lesional (p=0.0015) skin (**Figure 2A**). Conversely, α-diversity did not differ between non-lesional and lesional skin at either the pre-TSEBT (p=0.23) or post-TSEBT (p=0.07) time points (**Figure 2A**).

Pre-TSEBT versus post-TSEBT β-diversity differed significantly for lesional (p=0.001) and for non-lesional skin (p=0.005). By comparison, lesional versus non-lesional skin β-diversity did not differ significantly before (p=0.50) or after (p=0.21) TSEBT exposure (**Figure 2B**).

Pre- versus post-local IR did not demonstrate significant differences in α- or β-diversity (**Figures 2C**, **D**). This was true for both lesional pre- versus post-local IR and non-lesional pre versus post-local IR. Importantly, non-lesional pre- versus post-local IR serves as an internal control as these sites were not exposed to IR.

### TSEBT increases the relative abundance of healthy commensal skin bacteria

**Figure 3:**
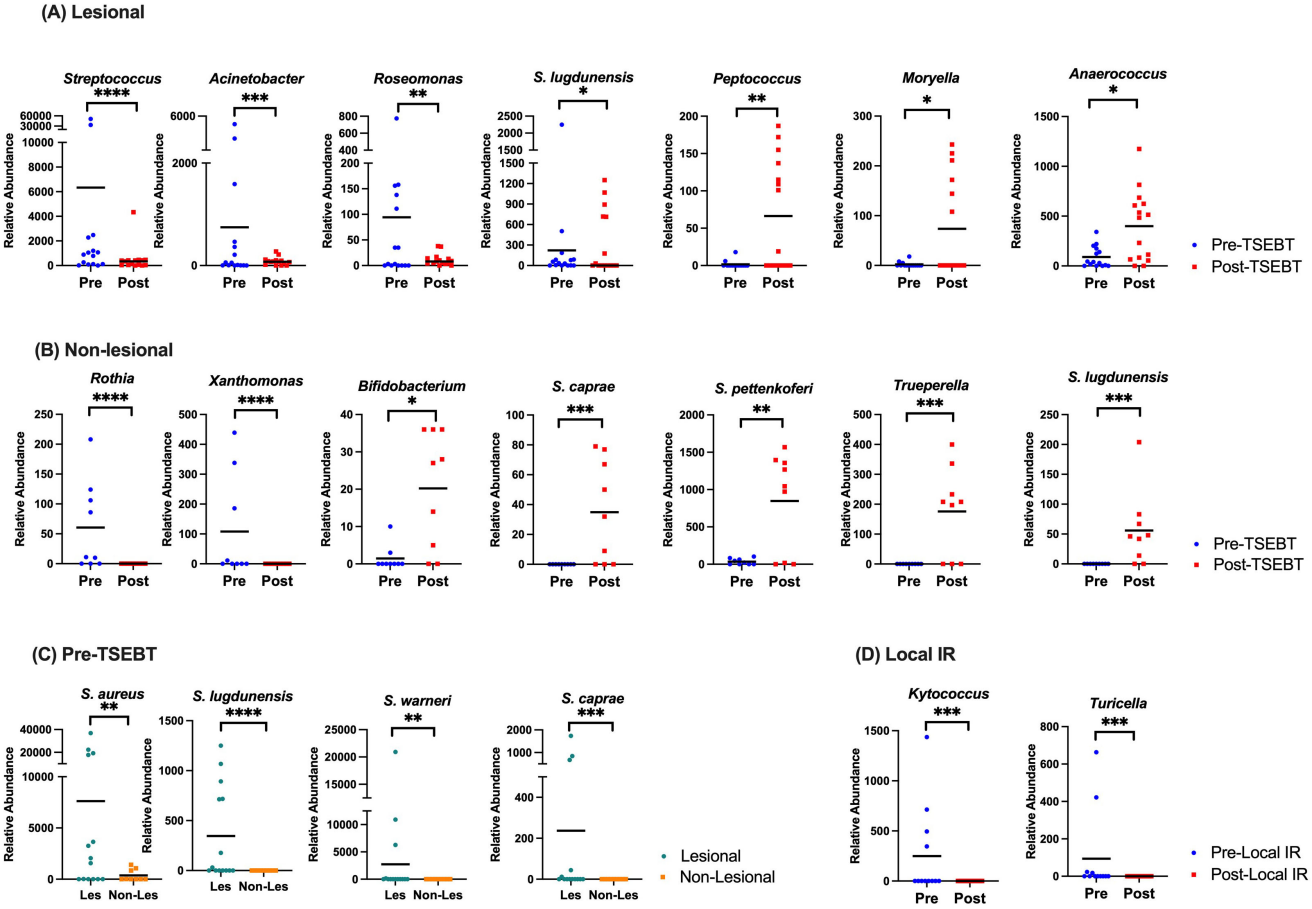
Taxon-by-taxon analyses amongst pre versus post-TSEBT skin and lesional versus non-lesional skin. **(A)** Analysis of lesional skin before TSEBT reveals increased relative abundance of *Streptococcus* and *Roseomonas* and other potentially pathogenic skin genera pre-TSEBT, and increases in the relative abundance of multiple anti-inflammatory taxa post-TSEBT. **(B)** Analysis of non-lesional skin reveals increased relative abundance of *Rothia* and other inflammatory taxa pre-TSEBT and increases in healthy commensals *Bifidobacterium*, *S. caprae*, and *S. pettenkoferi* post-TSEBT. **(C)** Species level *Staphylococcus* analyses of lesional versus non-lesional skin before TSEBT reveals increases in multiple taxa, including *S. aureus*. **(D)** Analysis of lesional skin reveals decrease in pathogenic *Kytococcus* and *Turicella* post-local IR compared to pre-local IR. The black line represents mean relative abundance. *p <0.05; **p<0.01; ***p<0.001; ****p<0.0001.

In lesional skin*, Streptococcus, Acinetobacter*, and *Roseomonas* were significantly less abundant post- versus pre-TSEBT (p<0.01, q<0.05) while *S. lugdunensis* trended lower (p<0.05, q>0.05). Healthy commensals including genera *Peptococcus* and *Cutibacterium*, and family *Anaerovoracaceae* were higher in post-TSEBT lesional skin (p<0.0001, q<0.0001), and genera *Moryella, Anaerococcus*, and *DNF00809*, a member of the *Eggerthellaceae* family, trended higher (**Figure 3A**). Other bacteria that increased post-TSEBT included *Trueperella*, *Olsenella*, and *Porphyromonas*.

In non-lesional skin, *Rothia, Xanthomonas, and Streptococcus* were increased pre-TSEBT relative to post-TSEBT (p<0.01, q<0.05). In contrast, *Bifidobacterium, S. caprae, S. pettenkoferi, S. lugdunensis*, *Trueperella* and several members of the order *Lactobacillales* were increased post-TSEBT relative to pre-TSEBT (p<0.01, q<0.05) (**Figure 3B**).

At the species level, *S. aureus, S. lugdunensis, S. warneri*, and *S. caprae* were significantly more abundant in pre-TSEBT lesional versus non-lesional skin (p<0.001, q<0.05) (**Figure 3C**). Post-TSEBT, there were no significant differences between lesional and non-lesional skin.

In treated lesions, *Kytococcus* and *Turicella* were lower post-local IR compared to pre-local IR (p<0.0001, q<0.0001) (**Figure 3D**).

### Healthy controls have significantly higher α-diversity than patients before, but not after, IR

**Figure 4:**
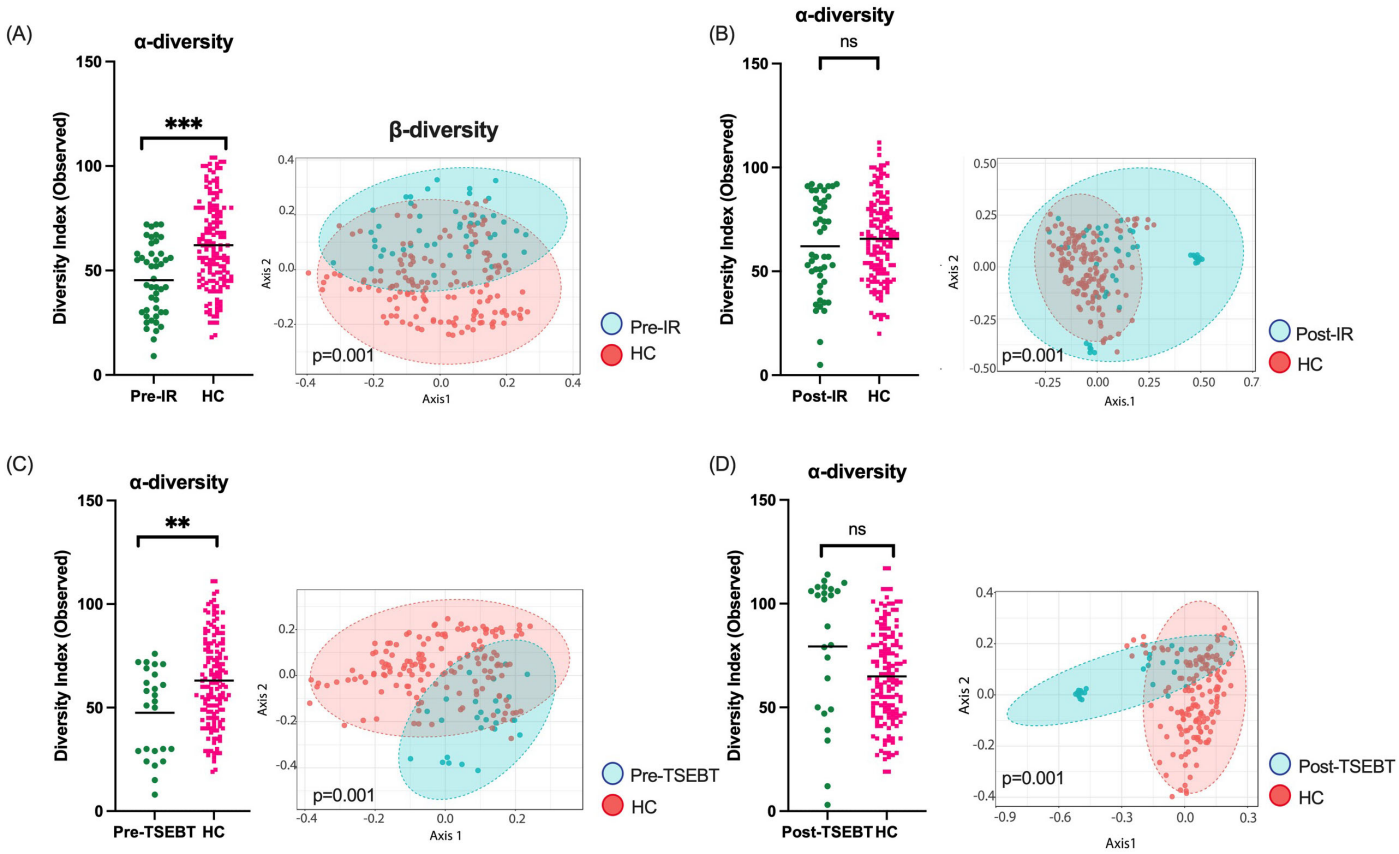
Healthy controls (HC) skin compared to CTCL skin before and after any form of IR and before and after TSEBT. **(A)** α-diversity was significantly lower in pre-IR patients than HC, and β-diversity differed significantly between the groups. **(B)** After any form of IR, CTCL skin α-diversity was no longer lower than that of HC, but β-diversities remained significantly different. **(C)** α-diversity was significantly lower in pre-TSEBT compared to HC skin, and β-diversity differed significantly between the groups. **(D)** Post-TSEBT α-diversity was no longer significantly different compared to HC but β-diversity remained different between post-TSEBT patients and HC. **p<0.01; ***p<0.001; ns, not significant.

Finally, we compared CTCL skin before and after any form of IR to matched HC skin. HC had significantly higher α-diversity compared to CTCL pre-IR (Observed p<0.0001), and microbial communities differed significantly (p=0.001) (**Figure 4A**). Relative abundances of *S. aureus* and *S. haemolyticus* were increased in CTCL patients, while *S. hominis, Bifidobacterium*, and several members of the order *Lactobacillales* were higher in HC. After IR, α-diversity no longer differed between HC and CTCL patients (p=0.53), whereas microbial communities remained distinct (p=0.001) (**Figure 4B**). DeSeq2 identified increased relative abundance of *S. aureus*, *S. caprae*, *S. haemolyticus*, and *S. lugdunensis* amongst CTCL patients. Comparison of pre-TSEBT patients versus HC and post-TSEBT versus HC revealed similar results to those seen in pre-IR versus HC and post-IR versus HC (**Figures 4C**, **D**).

## Discussion

We utilized 16S rRNA and *tuf* amplicon sequencing to explore changes to the skin microbiome after IR. Having previously established changes to the CTCL microbiome with exposure to non-ionizing radiation (i.e. nbUVB), we sought to describe the relationship between the CTCL microbiome and IR (10, 11). To our knowledge, this is the first study to longitudinally describe the skin microbiome of CTCL patients before and after IR. Our results support our hypothesis that shifts in microbial communities reflect post-IR lesion improvement.

We previously demonstrated increased α-diversity in the skin of CTCL patients who responded to nbUVB therapy (10). Regardless of whether ionizing or non-ionizing, CTCL skin improvement with total body irradiation appears to increase α-diversity. Furthermore, given that all study patients improved after IR (average mSWAT change -25.3), we expected to see similar changes to those observed in nbUVB responders. *Anaerococcus*, which reduces *S. aureus* growth and maintains skin homeostasis (32), and *S. pettenkoferi*, a healthy skin commensal, tracked higher in post-TSEBT lesional skin and were both increased amongst post-nbUVB responders (10, 33). Furthermore, *S. aureus* appears to decrease with response to both IR and nbUVB (10).

While the α-diversity of both lesional and non-lesional skin increased following TSEBT, this finding was not replicated in lesions targeted with local IR. In TSEBT, electron beams penetrate the entire skin surface (34). Local IR, given its targeted nature, may not have as strong or as lasting of an effect. A small amount of radiation to a targeted location is likely unable to overcome the homeostasis maintained by the microbial community existing across the entire skin surface (35). At either the pre- or post-TSEBT timepoints, α-diversity did not differ between lesional and non-lesional skin, similar to prior CTCL microbiome studies. This is consistent with the paradigm that skin dysbiosis in inflammatory skin diseases manifests at both lesional and non-lesional sites (7, 36, 37).

For both skin types, the relative abundance of genera implicated in skin infection, including *Streptococcus, Acinetobacter, Xanthomonas*, and *Rothia*, were decreased post-TSEBT versus pre-TSEBT (38–41). A notable increase following TSEBT includes *Moryella*. *Moryella* is a member of the *Lachnospiraceae* family, which produces butyrate – a short chain fatty acid with potent histone deacetylase inhibitor activity, the mechanism of two FDA-approved treatments for CTCL (42). Furthermore, in a study examining radiation-induced skin injury, *Lachnospiraceae* correlated with rapid healing of radiation injuries as opposed to chronic ulcer formation (16). At the species level, *S. aureus*, a known potentiator of CTCL, was significantly higher in pre-TSEBT lesional skin compared to non-lesional skin, but this was not the case post-TSEBT (3, 4, 43). Notably, few potential opportunistic pathogens, such as *Trueperella* and *Porphyromonas*, were increased post-IR and post-TSEBT skin. However, these shifts were relatively few in comparison to reduction of other pathogenic bacteria and increase of healthy commensals. As microbial richness is recouped in both lesional and non-lesional skin after TSEBT, this taxonomic shift may reflect attenuation of pathogenic bacteria like *Staphylococcus* in favor of communities enriched with protective and anti-inflammatory taxa like *Moryella*. These findings also suggest that TSEBT does not drive additional skin dysbiosis in CTCL or create a persistent microbe-barren environment but rather allows for recolonization of commensal bacteria associated with healthy individuals.

When analyzing all CTCL skin exposed to any form of IR, α-diversity was significantly higher post-IR. However, when only analyzing lesional skin exposed to any form of IR, increased α-diversity was not observed. As such, changes in non-lesional CTCL skin likely account for the overall increase in α-diversity observed after IR. This conclusion is supported by our findings that in non-lesional skin exposed to IR, α-diversity was significantly higher post-IR. It is possible that the microbiome of lesional skin may take longer to repopulate healthy bacteria than non-lesional skin, as non-lesional CTCL skin may have milder dysbiosis. Post-IR collections were completed, on average, 30 days after treatment. Thus, it is conceivable that had collections occurred at time points further out from IR, α-diversity may have been higher in post-IR lesional skin when compared to pre-IR lesional skin. Genera enriched after IR exposure included *Moryella* and *Bifidobacterium*. It has been suggested that *Bifidobacterium* may suppress the Th2 phenotype and improve skin barrier function in an AD skin model (44, 45). Notably, CTCL is also driven by a Th2 phenotype (46). Additionally, the trend towards lower *S. aureus* abundance may also allow for healthy commensal recolonization.

Lastly, our results demonstrated HC had significantly higher α-diversity than CTCL patients before any form of IR. However, post-IR, α-diversity no longer differed between the two groups. These results suggest that in part, successful IR treatment may result from normalization of the CTCL skin microbiome towards that of HC.

A limitation of this study includes its relatively small sample size. However, longitudinal analyses of patients given a common intervention eliminates inter-individual variability and provides a far more robust dataset compared to typical cross-sectional datasets for determining microbes associated with disease progression and response. To reduce cofounders, our cohort was well-characterized and rigorously controlled for factors outside of IR which may impact the CTCL microbiome. We excluded patients who used any form of antibiotics within the prior 4 weeks. Moreover, we surveyed for potential confounders including differences in diet, co-morbidities, and associated medications and there were no significant differences between CTCL patients and age-matched healthy controls. In the future, stricter control of potential confounders including hygiene practices and environmental exposures such as ultraviolet radiation may improve study validity.

This work further supports increasing evidence that the host microbiome is closely associated with CTCL pathogenesis and treatment response. Future studies include exploring changes to the gut microbiome with IR therapy, and studying whether altering the skin microbiota pre-radiation may contribute to increased local control of individual lesions and enhanced abscopal responses. Elucidating the timeline on which microbial shifts occur will also be essential to fully understanding this relationship. Finally, larger studies with longer follow-up will increase the robustness of our results and allow us to explore whether microbial shifts are sustained over time. In demonstrating decreased pathogenic, and increased protective microbial species with IR, this work also contributes to increasing evidence that the skin microbiome mirrors CTCL disease severity. Furthermore, while IR is known to work through its direct apoptotic effects, our work suggests that IR may also help restore the skin microbiome through the renewal of healthy commensals, and reduction of pathogenic microbes that may directly or indirectly drive antigenic stimulation of malignant T-cells. In the future, better understanding of the relationship between the skin microbiome and IR may allow for microbially-optimized IR treatment strategies that maximize tumor clearance and systemic anti-tumor immunity.

## Data Availability

The data presented in the study are deposited in the NCBI short read archive, accession number PRJNA1100592.
