# Supplementary material for: Ionizing radiation improves skin bacterial dysbiosis in cutaneous T-cell lymphoma
Source: Front Immunol. 2024 Dec 24;15:1520214. doi: 10.3389/fimmu.2024.1520214 (PMC11703887; doi:10.3389/fimmu.2024.1520214)
Supplement: Supplementary file 1 [file Table1.docx]

**Supplementary Table S1.** Patient demographics and medical history.

| **Sex** | **Age (years)** | **Race** | **FST** | **CTCL Subtype** | **Stage** | **Other Treatments** | **Comorbidities** | **non-CTCL Medications** |
| --- | --- | --- | --- | --- | --- | --- | --- | --- |
| Female | 71 | White | 2 | Mycosis Fungoides | IIB | Topical Steroids, NBUVB, Bexarotene | HTN, HLD | Levothyroxine, Lisinopril, Rosuvastatin |
| Male | 60 | White | 1 | PCAETCL |  | Topical Steroids, Bexarotene, Interferon | HCL, HTN, Hypothyroidism | Levothyroxine, Simvastatin, Enalapril |
| Male | 62 | White | 2 | Follicular Mycosis Fungoides | IA | None | GERD | Lansoprazole, Levothyroxine |
| Male | 70 | White | 2 | Mycosis Fungoides | IIB | Topical Steroids, Acitretin, Interferon | HTN, HCL, CKD | Amlodipine, Aspirin, Fenofibrate, Levothyroxine, Valsartan-HCTZ |
| Female | 77 | White | 2 | Folliculotropic Mycosis Fungoides | IA | Topical Steroids, Acitretin | HLD, HTN, GERD | Atorvaststin, Metoprolol, Omeprazole |
| Male | 21 | Black | 5 | Mycosis Fungoides | IIIA | Topical Steroids, Bexarotene | None | None |
| Male | 67 | Black | 5 | Mycosis Fungoides | IIB | Topical Steroids, Acitretin, Interferon | Diabetes | Atorvastatin, Dapagliflozin, Metformin, Dulaglutide |
| Male | 39 | White | 1 | Mycosis Fungoides | IIA | Topical Steroids | None | None |
| Male | 41 | White | 2 | Primary Cutaneous CD4+ small/medium T-cell lymphoproliferative disorder | IA | Clobetasol | HLD | Rosuvastatin |
| Male | 59 | Black | 5 | Mycosis Fungoides | IB | Triamcinolone ointment; Bexarotene | HTN, Hypothyroidism | Valsartan-HCTZ, Atorvastatin,  Levothyroxine |
| Male | 40 | Black | 5 | Mycosis Fungoides | IIB | Clobetasol, Triamcinolone, Romidepsin | None | None |
| Female | 67 | Black | 5 | Mycosis Fungoides | IIIA | Clobetasol | CAD, HTN | Losartan-HCTZ, ASA, Atorvastatin |

ASA: Aspirin; CKD: chronic kidney disease; CTCL: cutaneous T cell lymphoma; GERD: gastroesophageal reflux disease; HCTZ: hydrochlorothiazide; FST: Fitzpatrick skin type; HCL: hypercholesterolemia; HLD: hyperlipidemia; HTN: hypertension; nbUVB: narrowband ultraviolet B.

**Supplementary Table S2**. Ionizing radiation (IR) treatment details and time between treatment and pre- and post-IR collections.

| **Radiation Type** | **Pre-IR Collection to IR (days)** | **IR to Post-IR Collection (days)** | **Treatment details** |
| --- | --- | --- | --- |
| Local | 48 | 39 | 7 Gy over 1 fraction (4 locations) |
| TSEBT | 31 | NA | 40 Gy over 16 fractions |
| Local | 22 | NA | 4 Gy over 2 fractions (9 locations) |
| Local | 15 | 34 | 7 Gy over 1 fraction (1 location) |
| TSEBT | 20 | 21 | 12 Gy over 6 fractions with 8 Gy boosts over 4 fractions to 4 locations |
| TSEBT | NA | 47 | 12 Gy over 6 fractions |
| TSEBT | 40 | NA | 12 Gy over 7 fractions |
| Local | 24 | 10 | 7 Gy over 1 fraction (8 locations) |
| Local | 20 | 63 | 24 Gy over 12 fractions (1 location) |
| Local | 0 | 19; 109* | 8 Gy over 2 fractions (1 location) |
| TSEBT | 8 | 6; 126* | 12 Gy over 6 fractions with 8 Gy boosts over 4 fractions to 4 locations |
| TSEBT | 0 | NA | 6 Gy over 3 fractions with 8 Gy boosts over 2 fractions to 4 locations |

Gy: Gray; NA: not applicable; RT: radiation therapy; TSEBT: total skin electron beam therapy

*Two patients received two post-radiation collections, one shortly after their treatment, and one three months later.

**Supplementary Table S3.** Healthy control demographics and medications.

| **Sex** | **Age** | **Race** | **FST** | **Comorbidities** | **Concurrent Medications** |
| --- | --- | --- | --- | --- | --- |
| Female | 87 | White | 1 | None | None |
| Female | 79 | White | 3 | None | None |
| Female | 81 | White | 2 | None | None |
| Male | 86 | White | 3 | HTN, HLD, GERD | Amlodipine, Omeprazole, Simvastatin, Aspirin |
| Male | 26 | White | 1 | None | None |
| Male | 36 | White | 1 | None | None |
| Female | 59 | White | 2 | Asthma, HLD, hypothyroidism, GERD, Rheumatoid Arthritis | Albuterol, Atorvastatin, Levothyroxine, Pantoprazole, Methotrexate |
| Female | 68 | White | 3 | HLD | Atorvastatin |
| Male | 24 | Asian | 3 | None | None |
| Female | 27 | White | 4 | None | None |
| Female | 73 | White | 2 | HFrEF, HTN, HLD, DM, GERD, hypothyroidism | Carvedilol, Eplerenone, Esomeprazole, Furosemide, Insulin, Levothyroxine, Lisinopril, Rosuvastatin |
| Female | 57 | White | 2 | None | None |
| Male | 54 | White | 2 | GERD | Pantoprazole |
| Female | 65 | White | 2 | DM, GERD | Aspirin, Insulin Glargine, Semaglutide, Omeprazole |
| Female | 57 | White | 2 | None | None |
| Female | 73 | White | 2 | HLD, HTN, GERD | Losartan, Rosuvastatin, Omeprazole |
| Female | 33 | Asian | 3 | None | None |
| Male | 26 | Asian | 3 | None | None |
| Male | 37 | Other | 3 | GERD, HTN | Pantoprazole, Amlodipine |
| Male | 79 | White | 2 | HTN | Verapamil |
| Female | 57 | White | 2 | None | None |
| Male | 65 | White | 2 | HLD | Simvastatin |
| Male | 47 | White | 1 | None | None |
| Female | 23 | Asian | 3 | None | None |

DM: Diabetes Mellitus; FST: Fitzpatrick skin type; GERD: gastroesophageal reflux disease; HLD: hyperlipidemia; HTN: hypertension; HFrEF: heart failure with reduced ejection fraction.

**Supplementary Table S4.** Patient demographic and clinical characteristics.

|  | **Local IR** | **TSEBT** | **p-value** |
| --- | --- | --- | --- |
| **N** | 6 | 6 |  |
| **Mean age** (range), years | 57 (39-71) | 55 (21-77) | 0.87 |
| **Sex (%)**  Male  Female | 5 (83.3)  1 (16.7) | 4 (66.7)  2 (33.3) | 1.00 |
| **Race (%)**  White  Non-White | 5 (83.3)  1 (16.7) | 2 (33.3)  4 (66.7) | 0.24 |
| **FST (%)**  Light (I-III)  Dark (IV-VI) | 5 (83.3)  1 (16.7) | 2 (33.3)  4 (66.7) | 0.24 |
| **CTCL Subtype (%)**  Mycosis Fungoides  Other CTCL | 5 (83.3)  1 (16.7) | 5 (83.3)  1 (16.7) | 1.00 |
| **Stage (%)**  Early (IA-IIA)  Late (IIB-IVB) | 4 (66.7)  2 (33.3) | 1 (17)  5 (83) | 0.24 |
| **Non-RT treatments (%)**  Skin-directed only  Skin-directed & systemic  None | 2 (33.3)  3 (50.0)  1 (16.7) | 1 (16.7)  5 (83.3)  0 (0.0) | 0.55 |

CTCL (cutaneous T-cell lymphoma); FST (Fitzpatrick skin phototype); IR (Ionizing Radiation)

**Supplementary Table S5.** Patient and healthy control demographic and clinical characteristics.

| **N** | **HC (25)** | **Patients (12)** | **p-value** |
| --- | --- | --- | --- |
| **Mean age** (range), years | 56 (23-87) | 56 (21-77) | 0.96 |
| **Sex (%)**  Male  Female | 11 (44%)  14 (56%) | 9 (75%)  3 (25%) | 0.09 |
| **Race (%)**  White  Non-White | 20 (80%)  5 (20%) | 7 (58%)  5 (42%) | 0.24 |
| **Comorbidities (%)**  HTN  HLD  GERD  DM | 6 (24%)  7 (28%)  7 (28%)  2 (8%) | 4 (16%)  3 (12%)  2 (8%)  2 (8%) | 0.70  >0.99  0.69  0.58 |

DM (Diabetes Mellitus); GERD (gastroesophageal reflux disease); HC (healthy control); HLD (hyperlipidemia); HTN (hypertension)
